# Hydrogen Sulfide Impairs Meiosis Resumption in *Xenopus laevis* Oocytes

**DOI:** 10.3390/cells9010237

**Published:** 2020-01-17

**Authors:** Armance Gelaude, Sylvain Slaby, Katia Cailliau, Matthieu Marin, Arlette Lescuyer-Rousseau, Caroline Molinaro, Jan Nevoral, Veronica Kučerová-Chrpová, Marketa Sedmikova, Jaroslav Petr, Alain Martoriati, Jean-François Bodart

**Affiliations:** 1 CNRS, UMR 8576-UGSF-Unité de Glycobiologie Structurale et Fonctionnelle, Univ. Lille, F-59000 Lille, France; armance.gelaude@gmail.com (A.G.); sylvain.slaby@gmail.com (S.S.); katia.maggio@univ-lille.fr (K.C.); matthieu.marin@univ-lille.fr (M.M.); arlette.lescuyer@univ-lille.fr (A.L.-R.); caroline.molinaro@univ-lille.fr (C.M.); jean-francois.bodart@univ-lille.fr (J.-F.B.); 2 Department of Veterinary Sciences, Food and Natural Resources, Faculty of Agrobiology, Czech University of Life Sciences in Prague, Kamycka 129, 16521 Prague-Suchdol, Czech Republic; Nevoral.Jan@seznam.cz (J.N.); kucerova.chrpova@gmail.com (V.K.-C.); sedmikova@af.czu.cz (M.S.); 3 Biomedical Center and Department of Histology and Embryology, Faculty of Medicine in Pilsen, Charles University, Alej Svobody 1655/76, 32300 Pilsen, Czech Republic; 4 Research Institute of Animal Production, Pratelstvi 815, 10400 Prague 10-Uhrineves, Czech Republic; petr.jaroslav@vuzv.cz

**Keywords:** meiosis, hydrogen sulfide, *Xenopus laevis*, oocyte, cell cycle

## Abstract

The role of hydrogen sulfide (H_2_S) is addressed in *Xenopus laevis* oocytes. Three enzymes involved in H_2_S metabolism, cystathionine β-synthase, cystathionine γ-lyase, and 3-mercaptopyruvate sulfurtransferase, were detected in prophase I and metaphase II-arrested oocytes and drove an acceleration of oocyte meiosis resumption when inhibited. Moreover, meiosis resumption is associated with a significant decrease in endogenous H_2_S. On another hand, a dose-dependent inhibition was obtained using the H_2_S donor, NaHS (1 and 5 mM). NaHS impaired translation. NaHS did not induce the dissociation of the components of the M-phase promoting factor (MPF), cyclin B and Cdk1, nor directly impacted the MPF activity. However, the M-phase entry induced by microinjection of metaphase II MPF-containing cytoplasm was diminished, suggesting upstream components of the MPF auto-amplification loop were sensitive to H_2_S. Superoxide dismutase and catalase hindered the effects of NaHS, and this sensitivity was partially dependent on the production of reactive oxygen species (ROS). In contrast to other species, no apoptosis was promoted. These results suggest a contribution of H_2_S signaling in the timing of amphibian oocytes meiosis resumption.

## 1. Introduction

Hydrogen sulfide (H_2_S) is a gasotransmitter involved in different physiological and pathological processes, including inflammation, neuromodulation, regulation of vascular remodeling, protection against heart diseases, atherosclerosis, and pulmonary hypertension [1,2]. Endogenous H_2_S is produced by three enzymes: cystathionine-β-synthase (CBS), cystathionine-γ-lyase (CSE) and 3-mercaptopyruvate sulfurtransferase (MPST) [3,4,5]. CBS, CSE, and MPST are noticeably expressed in the reproductive organs of mice [6], rats [7], and humans [8]. It was supposed that the alteration of the H_2_S metabolism could lead to reproductive abnormalities but evidence was sparse [9,10]. Distinct distribution of CSE and CBS was reported in rat testis, where CSE was detected in Sertoli cells and CBS in Sertoli, Leydig, and germ cells, suggesting a testis function [7,11]. H_2_S was mainly involved in male erectile function in primates [12], rats [13], rabbits [14], and humans [15]. Moreover, a limited number of studies have addressed the impact of H_2_S on the female reproductive system. H_2_S was associated with an increase in the rate of spontaneous abortion [16]. A role of H_2_S at the physiological level in the female reproductive system exists. In rodents, CSE and CBS play different roles [17]. Knockout experiments underlined that while CSE invalidation led to no defects in female reproductive functions [18], CBS is necessary for female fertility [6]. CBS knockout was reported to drive a decrease in the uterine mass, a decrease in the follicle population [10], and an inhibition of oocyte maturation [19]. Nevertheless, H_2_S mechanisms of action during female oogenesis, oocytes maturation, and meiosis resumption remain poorly understood. Few studies have been performed on the direct impact of H_2_S on oocytes. Recently, aging in porcine oocytes was prevented by H_2_S donors [20]. Although CBS, CSE, and MPST were observed in porcine oocytes [21], CBS was not detected in mice oocytes [6]. In porcine oocytes, a direct effect of H_2_S was reported: H_2_S donors accelerated meiotic resumption while inhibitors of H_2_S metabolism prevented oocyte maturation [21,22].

In the present work, we evaluate the impact of the gasotransmitter H_2_S on the regulation of the meiosis progression in *Xenopus laevis* oocytes. We focused on the effects of H_2_S metabolism on meiosis resumption using an H_2_S donor and inhibitors. We took advantage of the amenability of this model to carry out a study in the context of both reproduction and cell cycle transition at the single-cell level. *Xenopus* oocytes have provided for many decades a pertinent model to study cell cycle progression and regulation. Blocked in prophase of the first meiotic division—in a state analogous to the G2 phase of mitosis—oocytes resume meiosis upon stimulation by progesterone addition. Considered as an M-phase entry, the meiotic resumption is characterized at the morphological level by the occurrence of a white spot at the cell apex, attesting for the migration and the dissolution of the germinal vesicle (germinal vesicle breakdown, GVBD). These oocytes progress from prophase I (PI) until metaphase II (MII) of meiosis, where they are blocked in anticipation of fertilization. During meiotic resumption, the migration of the nuclear material towards the animal pole is followed by the organization of a meiotic spindle. At the molecular level, the meiotic resumption is triggered through a non-genomic pathway by the activation of a universal factor, MPF (M-phase promoting factor, composed by the Cdk1/CyclinB complex), which is activated by the pivotal Cdc25c phosphatase [23,24]. In parallel, the activation of the MAPK/Erk, Erk2 or Xp42mpk1 cascade [25] is mandatory for proper maturation and the absence of DNA synthesis between the two meiotic divisions [26,27,28]. The MAPK/Erk protein level present in *Xenopus* oocytes does not change during meiosis [28] and exhibits an all-or-none, ultrasensitive and bistable activation response, which results from a positive feedback loop [29] also observed for the downstream relay p90^Rsk^ [30,31,32].

Our analyses emphasized that H_2_S natural production is decreased in *Xenopus* oocytes arrested in metaphase II (the M-phase of the cell cycle), before MPF activation, compared to prophase I blocked oocytes (G2 phase). Preventing H_2_S metabolism accelerated oocyte maturation, whereas the addition of NaHS, an H_2_S donor, impaired meiosis resumption. NaHS could block meiosis resumption in response to progesterone in two distinct ways: by negatively regulating protein synthesis and targeting the MPF auto-amplification loop on upstream regulatory targets such as phosphatase Cdc25C. We report that H_2_S modulates oocyte meiosis in amphibians, and discuss the action mechanisms of this gasotransmitter.

## 2. Materials and Methods

### 2.1. Reagents

All reagents were obtained from Sigma–Aldrich Chimie (Saint-Quentin Fallavier, France), except antibodies (Santa Cruz Biotechnology, Dallas, TX, USA; Abcam, Paris, France; Invitrogen Thermo Fisher Scientific, Waltham, MA, USA; and Cell Signaling, Danvers, MA, USA). All tested solutions and media were prepared daily (freshly) or obtained by appropriate dilutions from stock solutions in Nathan Dascal medium (ND96).

### 2.2. Frog and Oocyte Handling

After anesthesia of the *Xenopus laevis* females (purchased from the CRB-University of Rennes I, Rennes, France, and housed in PHExMAR–University of Lille) by immersion in 1 g/L MS222 solution (tricaine methane sulfonate), ovarian lobes were surgically removed and placed in ND96 medium (96 mM NaCl, 2 mM KCl, 1.8 mM CaCl_2_, 1 mM MgCl_2_, 5 mM HEPES-NaOH, pH 7.5). Fully grown stage VI oocytes were isolated and defolliculated by partial ovarian tissue digestion with a collagenase A treatment for 30 min (1 mg/mL) followed by a manual microdissection. Oocytes were stored at 14 °C in ND96 medium until required. All animal experiments were performed according to the rules of the European Community Council guidelines (86/609/EEC) for laboratory animal experimentation. The animal protocol was approved by the local institutional review board (Comité d’Ethique en Expérimentation Animale, Haut de France, F59-00913).

### 2.3. Oocyte treatments, mRNA Micro-Injections, and Meiotic Resumption Analysis

Meiotic resumption was induced by oocytes incubation in ND96 medium containing 4 μg/mL of progesterone. The maturation process was scored by the appearance of a white spot at the animal pole of the oocyte. Oocytes were pre-incubated 1 h with NaHS, a donor of H_2_S, at different concentrations (from 100 µM to 5 mM), AOAA (aminooxyacetic acid-10 mM), KGA (ketoglutaric acid-10 μM), PAG (dl-propargylglycine-10 μM) [33] prior progesterone addition. SOD (superoxide dismutase, 150 units) and/or catalase (80 units) were added for 1 h prior NaHS treatments. Myt-Myc and Shb-Myc mRNA were prepared as previously described [34,35] and micro-injected into the equatorial region of prophase I-arrested oocytes (0.25–1 mg/mL). Oocytes were placed at 19 °C. Oocytes displaying a white spot were individually scored each hour for 15 h (rise of the germinal vesicle at the animal pole). The time required to obtain 50% of mature oocytes (GVBD_50_) enables the comparison between the maturation kinetics of oocytes from at least three different females.

### 2.4. Mature Oocyte Cytoplasm Micro-Injections

Defolliculated oocytes were incubated overnight at 19 °C in the presence of progesterone (4 μg/mL). Mature oocytes with a white spot were selected and rinsed twice in ND96 medium. Forty nanoliters of cytoplasm were withdrawn from these donor oocytes, using a positive displacement digital micropipette (Nichiryo, Tokyo, Japan) and 10 nL were injected at the equatorial region of recipient immature oocytes in order to avoid injecting the germinal vesicle located in the animal hemisphere. Prior to the micro-injection process, immature oocytes were incubated for 1 h in NaHS (100 μM–5 mM). Micro-injected oocytes were placed at 19 °C and the white spot appearance was monitored hourly.

### 2.5. Heat Fixation and GVBD Analysis

After phenotypic analysis of the white spot appearance, oocytes were heated for 15 min at 100 °C. Oocytes were bisected along the animal/vegetative axis to seek the presence of the germinal vesicle.

### 2.6. Electrophoresis and Western blot

Each oocyte was lysed as a single cell in 10 µL of the following homogenization buffer (50 mM HEPES pH 7.4, 500 mM NaCl, 0.05% SDS, 0.5% Triton X100, 5 mM MgCl_2_, 1 mg/mL bovine serum albumin, 10 μg/mL leupeptin, 10 μg/mL aprotinin, 10 μg/mL soybean trypsin inhibitor, 10 μg/mL benz-amidine, 1 mM PMSF, 1 mM sodium vanadate) and centrifuged for 5 min at 10,000× *g* (4 °C). The supernatant was mixed with one volume of Laemmli 2X buffer added with 4% beta-mercaptoethanol, heated at 100 °C for 3 min and stored at −20 °C until analysis. Proteins were separated by SDS–PAGE (15–17%) and transferred onto nitrocellulose membranes (Hybond, Amersham Pharmacia Biotech Ltd, Buckinghamshire, United Kingdom). Membranes were blocked with 5% low fat dry milk and incubated with specific antibodies overnight. Polyclonal rabbit antibodies used where raised against p90^Rsk1^ (C-21 sc231, Santa Cruz Biotechnology Inc., Dallas, TX, United States; 1/1000), phosphorylated histone H3 (S10, Cell Signaling; 1/1500), MPST (Sigma Aldrich, Saint Quentin Fallavier, France; 1/1000), Cdk1 (17, Santa Cruz Biotechnology; 1/1500), and monoclonal mouse antibodies against Erk2 (D-2 sc1647, Santa Cruz Biotechnology; 1/3000), Tyr15 phosphorylated Cdk1 (tyr15, Cell Signaling; 1/1500), CSE (Sigma Aldrich; 1/1000), CBS (Sigma Aldrich; 1/1000), cyclin B2 (sc53239, Santa Cruz Biotechnology; 1/1500), Mos (c237, Santa Cruz Biotechnology; 1/500) and cytochrome C (7H8.2C12, ab13575, Abcam, Cambridge, United Kingdom; 1/1500). Nitrocellulose membranes with bound primary antibody were incubated with appropriate secondary antibodies (Sigma–Aldrich). The signals were detected using a chemiluminescent assay (ClarityTM Western ECL Substrate, Bio-Rad, Hercules, CA, United States). Bands were quantified by Image J software (version 1.52i, NIH, Bethesda, MD, USA) and means of 3 independent experiments were calculated.

### 2.7. Immunoprecipitations

Cdk1 immunoprecipitations were performed on 50 immature oocytes lysed in 200 µL of homogenization buffer. After centrifugation at 4 °C for 15 min at 10,000× *g*, the protein extracts were incubated with different concentrations of NaHS (0 µM, 500 µM, 1 mM, and 5 mM) for 2 h at 4 °C. Protein extracts were pre-washed in the presence of Protein A-sepharose beads (5 mg, Sigma) for 1 h at 4 °C. Anti-Cdk1 antibody (anti-Cdk1, 17, Santa Cruz Biotechnology; 1:200) was added overnight at 4 °C followed by A-sepharose beads (5 mg, Sigma) addition for 1 h at 4 °C. The immune complexes were collected by centrifugation (10,000× *g*, 1 min, 4 °C), rinsed 3 times and re-suspended in one volume of Laemmli 2X buffer. Proteins were separated on 12.5% SDS PAGE gels and analyzed by immunoblotting according to the Western blot protocol. Bands were quantified by Image J software (version 1.52i) and means of 3 independent experiments were calculated.

### 2.8. Histone H1 Kinase Assay

Assays were performed on protein extracts from 50 mature oocytes lysed in 200 µL of homogenization buffer. After centrifugation (10,000× *g*, 15 min, 4 °C), the protein phase was incubated with different concentrations of NaHS (100 µM, 500 µM, 1 mM, and 5 mM) for 24 h at 4 °C. Samples were immediately frozen and stored at −80 °C until analysis. The kinase activity assay reaction of MPF, based on its capacity to phosphorylate external specific substrate, histone H1, was initiated by addition of 5 µL of buffer consisting of 100 mM 3-[*n*-morpholino] propanesulfonic acid pH 7.2, 20 mM para-nitrophenyl phosphate, 40 mM β-glycerolphosphate, 20 mM MgCl_2_, 10 mM EGTA, 0.2 mM EDTA, 5 µM cAMP-dependent protein kinase inhibitor, 2 mM benzamidine, 40 µg/mL leupeptin, 40 µg/mL aprotinin, 600 µM ATP, added with 2 mg H1/mL, and 500 µCi/mL [γ-^32^P]ATP (GE Healthcare Life Sciences, Little Chalfont, United Kingdom). Reactions were conducted for 30 min at 30 °C and blocked by the addition of one volume of Laemmli 2X buffer before they were boiled for 3 min. After electrophoresis, the 15% SDS PAGE gels were stained with Coomasie Blue R250 (Sigma–Aldrich Chimie), destained overnight, dried, and autoradiographed. Phosphorylated histone H1 signals were visualized by MultiGauge 2.0 software. Bands were quantified using Image J software (version 1.52i) and means of 3 independent experiments were calculated.

### 2.9. Apoptosis Analysis

Oocytes, stimulated overnight by progesterone, were treated with or without 5 mM NaHS at room temperature for 52 h [36]. While maturation completion can be obtained within 12–16 h, apoptosis phenotypes were analyzed throughout 72 h. Proteins were extracted at 8, 24, 48, 56, and 72 h, and then separated on 12.5% SDS PAGE gels and analyzed by immunoblotting using anti Erk2, anti-Cyclin B2, anti-p90^Rsk^, and anti-cytochrome C antibodies.

### 2.10. H_2_S Production

For each untreated or NaHS treated condition, ten oocytes were lysed at 4 °C in 5 μL pyridoxal 5-phosphate (0.2 M), 50 μL L-cystein (10 mM), 445 μL deionized water, and 250 μL of zinc acetate (1%), as described [20]. The reaction was started at 37 °C for 1 h and stopped by the addition of 250 μL of 50% trichloroacetic acid for 1 h at 37 °C. Samples were completed by addition of 133 μL *N*,*N* dimethyl-*p*-phenylenediamine sulphate (20 mM in 7.2 M HCl) and 133 μL FeCl3 (30 mM in 1.2 M HCl) to form methylene blue. The supernatants were collected after centrifugation at 10,000× *g* for 15 min. The absorbance was measured at 670 nm in a microplate reader (SPECTROstar Nano, BMG Labtech, Thermo Fisher Scientific, Waltham, MA, United States). Results are shown as relative ratios to prophase I oocytes.

### 2.11. Cdc25C Phosphatase Activity Assay

The reactions were performed in 500 µL of assay buffer (100 mM Tris-HCl, 40 mM NaCl, 1 mM DTT, 20% glycerol, pH 8.2) containing 1 µg of human recombinant Cdc25C (Sigma–Aldrich) and pre-incubated or not for 15 min at room temperature with NaHS (500 μM, 1 mM, or 5 mM). The reactions (15 min at 37 °C in the dark) are started by the addition of 3-OMFP (3-O-methylfluoresceinphosphate) (Sigma–Aldrich) to a final concentration of 500 μM. The fluorescence intensity that results from the transformation of 3-OMFP into 3-OMF (3-O–methylfluorescein) by Cdc25C phosphatase is measured at 520 nm after excitation at 480 nm. Results are shown as relative ratios to control without NaHS.

### 2.12. Statistical Analysis

All results are shown as mean +/− standard error of the mean (SEM); N refers to the number of separate experiments performed (number of females) while n is the number of treated oocytes. Experiments were performed at least in triplicate and on three different females. Significant differences were assessed with SigmaStat 3.1 software (SysStat, Erkrath, Germany) by means of one-way ANOVA followed by posthoc Tukey’s tests, except for H_2_S production and the Cdc25C activity assay; they were assessed with a Dunnett test. Statistical significance was accepted for * *p* < 0.05, ** *p* < 0.01, and *** *p* < 0.001.

## 3. Results

### 3.1. NaHS, an H_2_S Donor, Prevents the Xenopus Oocytes Meiotic Resumption in a Dose-Dependent Manner

**Figure 1:**
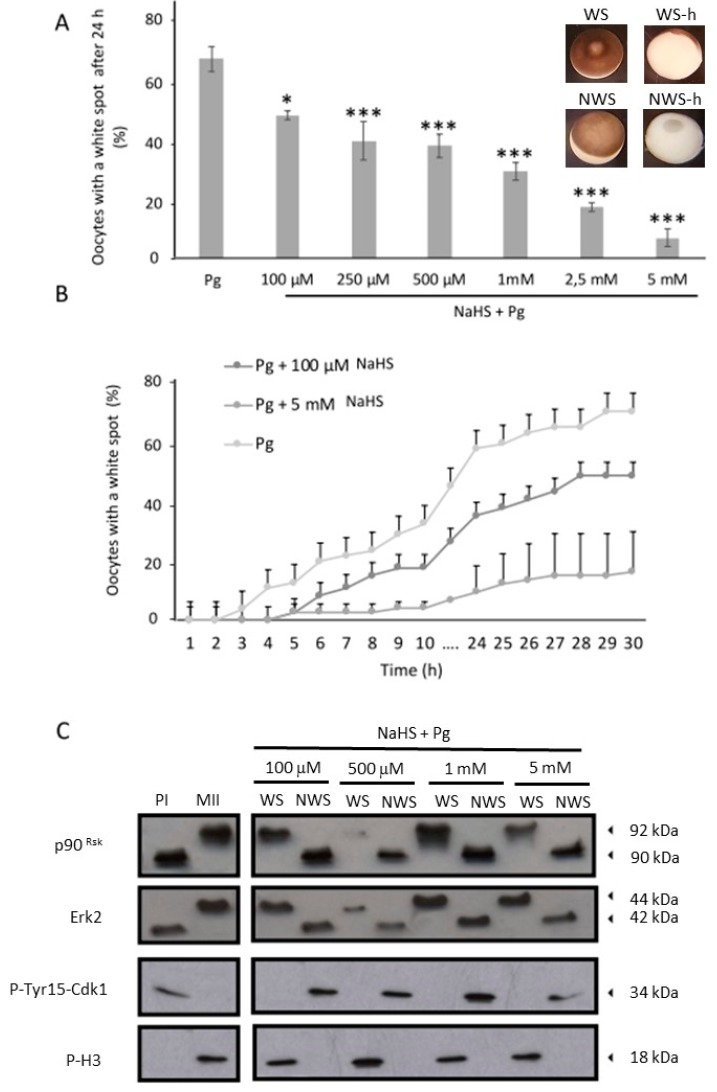
NaHS inhibits meiosis resumption induced by progesterone in a dose-dependent manner. Oocytes were preincubated in NaHS at different concentrations before progesterone addition (4 μg/mL). (**A**) Percentage of oocytes with a white spot 24 h after addition of progesterone. Statistical significance between control and NaHS conditions was accepted for * *p* < 0.05 and *** *p* < 0.001. Microphotographic of whole oocytes with (WS) or without (NWS) white spots and of bisected oocytes along the animal/vegetative axis after heating 15 min at 100 °C (WS-h/NWS-h) are shown. (**B**) Oocytes exhibiting white spots were scored every hour (0–10 h and 24 h–30 h). (**C**) Western blot of p90^Rsk^ and Erk2 (on modified polyacrylamide gels, the phosphorylated forms have a slower mobility shift), phospho-Tyr15-Cdk1 and phospho-ser10-H3. Some oocytes were maintained in culture medium without treatment (prophase I, PI) or treated by progesterone alone (metaphase II, MII). Oocytes were sorted into two groups: without (NWS) or with a white spot (WS). Experiments were performed on 20 oocytes per condition and three independent females.

Increasing NaHS concentrations ranging from 100 µM to 5 mM were applied. Even at the lowest concentrations, NaHS led to a significant decrease in oocytes resuming meiosis, as attested by the decrease of white spot occurrence rates (Figure 1A). The effect of NaHS on meiosis resumption is dose-dependent (Figure 1A). In the control condition, 65% of the oocytes showed a maturation spot against 48% and 10% in the presence of, respectively, 100 µM (*p* < 0.05) and 5 mM (*p* < 0.001) of NaHS (Figure 1A). Moreover, the kinetic experiment showed that the NaHS treatment delayed the meiosis resumption by 2 h (Figure 1B).

To confirm that oocytes without white spots were blocked in prophase I, the phosphorylation status of Erk2, p90^Rsk^ (downstream Erk2 effector), Cdk1, and Histone H3 (downstream target of MPF) were analyzed by Western blotting at the single-cell level. Erk2 and p90^Rsk^ were analyzed on modified acrylamide gels where the dephosphorylated forms have a faster mobility shift compared to the phosphorylated forms, while Cdk1 and Histone H3 were analyzed using antibodies directed against the phosphorylated forms. Prophase I oocytes exhibited phosphorylation of Cdk1 on tyrosine 15 and the absence of phosphorylated histone H3, Erk2, and p90^Rsk^. On the contrary, mature oocytes showed phosphorylation of histone H3, Erk2 and p90^Rsk^ and dephosphorylation of Cdk1 on tyrosine 15 (Figure 1C). Thus, two separate populations were discriminated: a population of immature oocytes that did not exhibit white spot-cells still arrested at prophase I (diplotene stage) and a mature oocyte population exhibiting white spots.

### 3.2. Inhibition of H_2_S Metabolism Key Enzymes Accelerates Oocyte Meiotic Resumption

**Figure 2:**
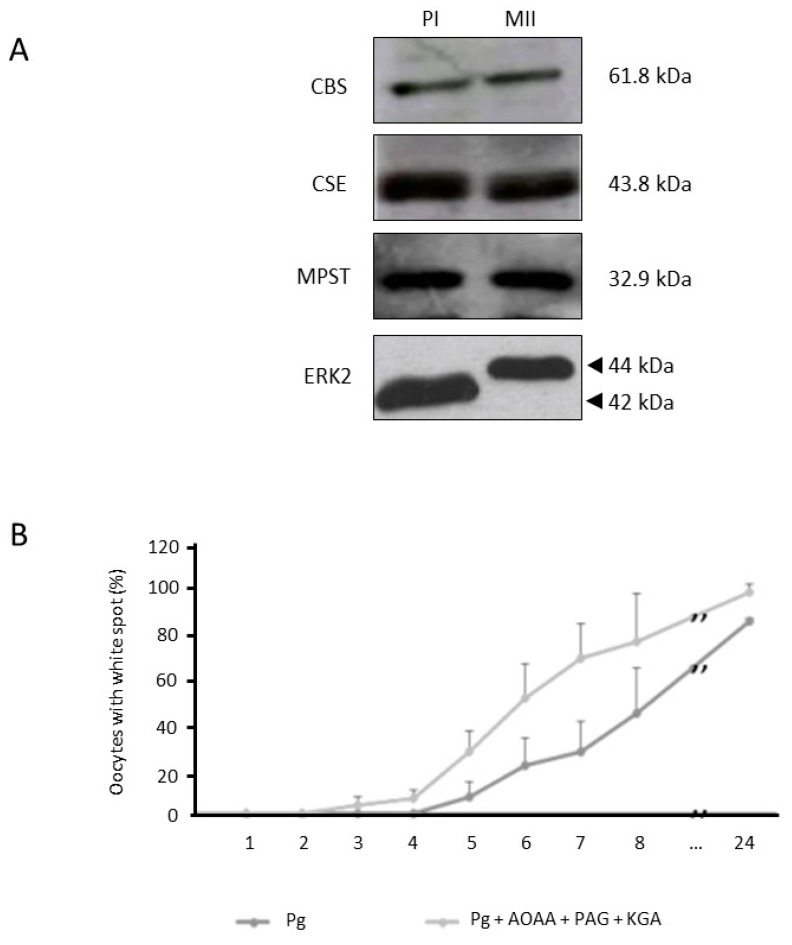

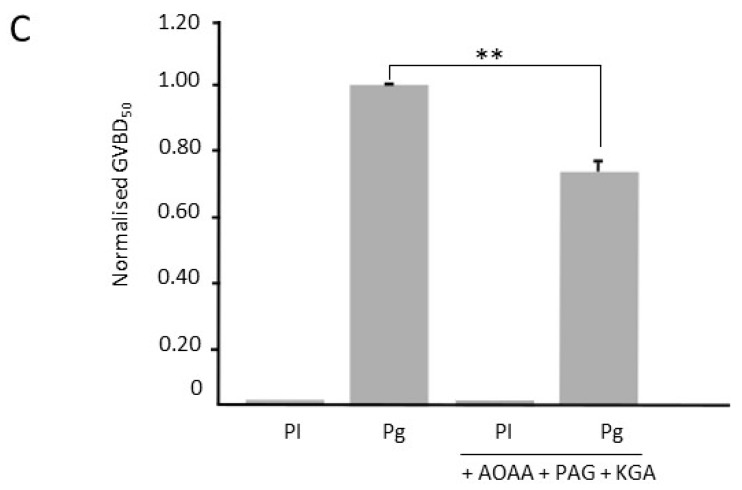
Inhibitors of H_2_S-releasing enzymes accelerate meiosis resumption. (**A**) Western blot detection of endogenous enzymes of the H_2_S metabolism, CBS, CSE, and MPST, in untreated oocytes (PI) and oocytes treated by progesterone (4 μg/mL) (MII). Total phosphorylated and unphosphorylated forms of Erk2 serves as loading controls. (**B**) Oocytes were pre-incubated or not with a cocktail of H_2_S-releasing enzymes inhibitors AOAA (aminooxyacetic acid-10 mM), PAG (dl-propargylglycine-10 μM), and KGA (ketoglutaric acid-10 μM) before progesterone (4 μg/mL) stimulation (Pg + AOAA + PAG + KGA) and their white spots were recorded every hour for 24 h. (**C**) GVBD_50_ corresponded to the time required to obtain 50% of mature oocytes. GVBD_50_ were normalized with the GVBD_50_ of the oocytes treated with progesterone only. Twenty oocytes were used in each condition from 3 independent females. Statistical significance was accepted for ** *p* < 0.01.

We further detected in *Xenopus* oocytes the presence of the three enzymes involved in the endogenous H_2_S metabolism, cystathionine β-synthase (CBS), cystathionine γ-lyase (CSE), and 3-mercaptopyruvate sulfurtransferase (MPST) in immature prophase I (PI) and mature metaphase II blocked (MII) oocytes (Figure 2A). CBS, CSE, and MPST were respectively impaired with appropriate specific chemical inhibitors, AOAA, PAG, and KGA. Seven hours after the incubation of oocytes with progesterone and the three H_2_S metabolism inhibitors, 70% of the oocytes present a maturation spot against 30% in the control condition (Figure 2B). Mean values of the GVBD_50_, for 3 females, indicated a significant acceleration of the maturation process since control oocytes reached 50% of GVBD (GVBD_50_ = 1) in an average of 14 h against 9 h in the presence of inhibitors (GVBD_50_ = 0.64) (*p* < 0.01) (Figure 2C). No GVBD was observed in oocytes without progesterone treatment pre-incubated or not with chemical inhibitors AOAA, PAG, and KGA (Figure 2C). The use of the inhibitors one by one or two by two was not efficient to facilitate GVBD induced by progesterone. The H_2_S metabolism inhibition and therefore the absence of newly produced H_2_S in *Xenopus* oocytes accelerated meiotic resumption.

### 3.3. Endogenous H_2_S Is Produced in Xenopus Oocytes

**Figure 3:**
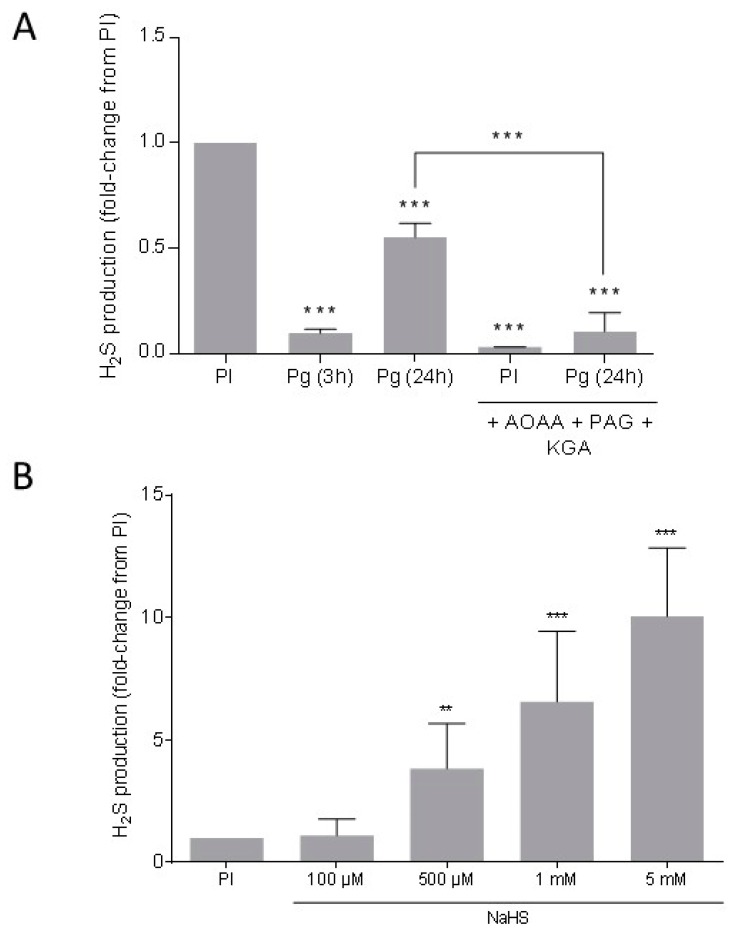
H_2_S production in *Xenopus* oocytes. (**A**) Endogenous H_2_S measurement was performed in prophase 1 blocked oocytes (PI) and oocytes 3 or 24 h after progesterone (4 μg/mL) (Pg 3 h or 24 h) addition without or with a 1 h pre-incubation with three inhibitors of H_2_S metabolism (AOAA + PAG + KGA). (**B**) Exogenous production of H_2_S was detected in oocytes after a pre-incubation of 1 h with a NaHS donor at 100 μM, 500 μM, 1 mM, and 5 mM. For each condition, four independent experiments were performed with n = 40. Statistical significance was accepted for ** *p* < 0.01 and *** *p* < 0.001.

An enzymatically and natural production of H_2_S was detected in oocytes using a colorimetric method based on the conversion of *N*,*N*-dimethyl-*p*-phenylenediamine to methylene blue directly in the presence of an oxidizing agent (acidified ferric chloride). Results are shown as relative ratios to prophase I oocytes H_2_S production. The production of endogenous H_2_S was decreased by 6.1-fold in oocytes 3 h after progesterone addition and by 1.8-fold in metaphase II oocytes 24 h after treatment by progesterone. The inhibition of the three H_2_S producing enzyme activities by specific chemical inhibitors, AOAA, PAG and KGA, in PI and Pg treated oocytes was significantly reduced (*p* < 0.001) compared to their respective untreated controls (Figure 3A). Oocytes submitted to a 1 h pre-incubation with the NaHS donor significantly raised their H_2_S production by 3.8-, 6.5-, and 10-fold when treated respectively by concentrations of 500 µM, 1 mM, and 5 mM (Figure 3B). No significant difference was observed between H_2_S productions of control oocytes and exposed oocytes to 100 µM of NaHS.

### 3.4. NaHS Impairs Oocyte Protein Synthesis

**Figure 4:**
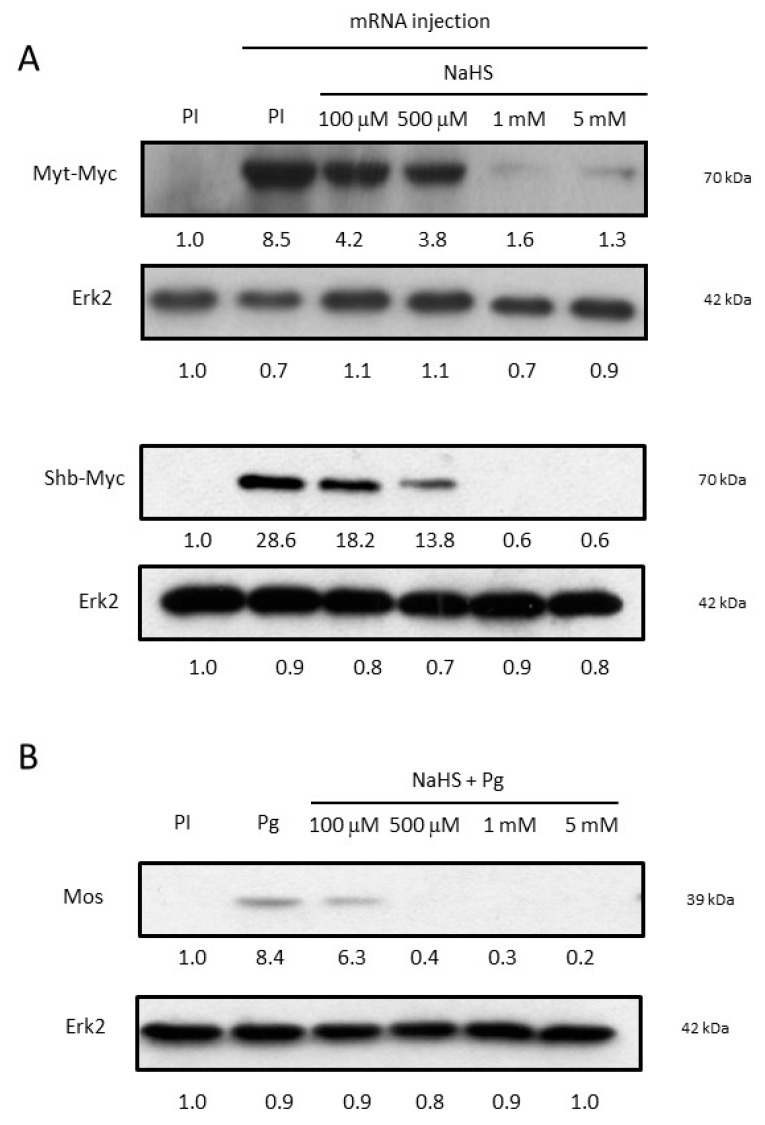
NaHS prevents protein synthesis. (**A**) Western blot of Myt-Myc and Shb-Myc tagged proteins, and Erk2 from immature oocytes (PI) or oocytes micro-injected with mRNA coding for a Myc tagged proteins after pre-incubation or not for 15 min in NaHS at different concentrations. (**B**) Western blot of endogenous Mos and Erk2 from ten pooled oocytes 15 h after NaHS treatments and progesterone induction (4 μg/mL). Relative intensity of Myc and Mos bands were normalized with the intensity of their respective loading control bands of Erk2. Myc, Mos, and Erk2 band intensity were estimated using Image J software.

*Xenopus* oocytes translate heterologous mRNA when injected in their cytoplasm under proper conditions [37,38]. Myt-Myc and Shb-Myc mRNA [34,35] were micro-injected into oocytes pre-incubated during 15 min in NaHS at increasing concentrations (100 µM up to 5 mM). Translation of the tagged-protein was assessed by Western blotting after 8 h. In the micro-injected oocytes, Myt and Shb mRNA were translated into protein (Figure 4A). Myt is inactivated by newly produced MPF when oocytes are exposed to progesterone and Shb adaptor protein is active only when receptor tyrosine kinase is stimulated by growth factors. The two Myt and Shb mRNA were used here in conditions where their protein remained inert, without progesterone or growth factor stimulation. Erk2, present at a constant concentration in *Xenopus* oocytes, was used as a loading control. The Myt-Myc and the Shb-Myc proteins were absent from the not-injected control oocytes, as expected. In the presence of the highest concentrations of NaHS (1 and 5 mM), the translated Myc proteins were reduced (Figure 4A). We observed a decreased of Myt-Myc protein levels of 50% and Shb-Myc to 36% and 51% for the lowest concentrations of NaHS (100 and 500 µM), and a decreased of 90% of Myt-Myc protein and 98% of Shb-Myc protein levels with the highest concentrations (1 and 5 mM). Furthermore, NaHS prevented the translation of endogenous Mos mRNA (Figure 4B) in *Xenopus* oocytes treated by NaHS at concentrations of 500 μM, 1 mM, and 5 mM. We could, therefore, suppose that H_2_S could interfere with protein synthesis necessary for oocyte meiotic resumption.

### 3.5. NaHS Impairs the Self-Amplification Loop of MPF

**Figure 5:**
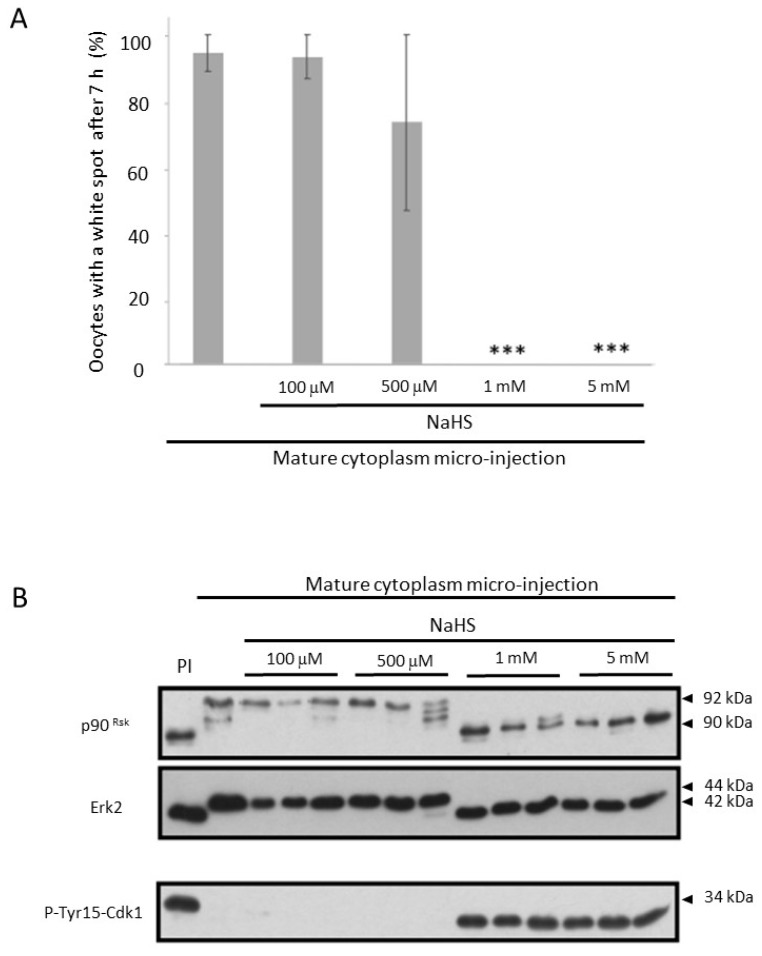
High concentrations of NaHS inhibit meiosis resumption induced by micro-injection of mature oocyte cytoplasm. Immature oocytes were pre-incubated (or not) in NaHS at different concentrations for 1 h before microinjection of mature cytoplasms. Some oocytes were maintained in culture medium without treatment (PI). (**A**) Percentage of oocytes exhibiting a white spot 7 h after micro-injection of mature oocyte cytoplasm. Statistical significance was accepted for *** *p* < 0.001. N = 3; n = 15 (**B**) Western blot analysis of p90^Rsk^, Erk2, and P-Tyr15-Cdk1 for three oocytes/NaHS conditions.

Then we tested whether NaHS could impair meiosis through an effect on the MPF auto-amplification loop. During the process of meiosis resumption, a small amount of active Cdk1 is generated by translation of the cyclin B pathway, which initiates the MPF auto-amplification loop. This loop relies on Cdk1 activity that regulates by phosphorylation its own regulators, Myt1 and Cdc25. Myt1 is inhibited whereas Cdc25 is activated. As a result, the inactive stock of Cdk1-cyclin B complexes is dephosphorylated at T14 and Y15 and fully activated to allow meiosis re-entry, a process visualized in *Xenopus* oocytes by the nuclear envelop breakdown (GVBD). Micro-injections of mature oocyte cytoplasm into immature oocytes exposed to NaHS were performed. Under these conditions, NaHS concentrations superior to 500 µM led to a total inhibition of the induced meiotic resumption (Figure 5A). In micro-injected control oocytes, without NaHS exposure, 94% of the oocytes showed a white spot. In a similar manner, 93% and 73% of the oocytes had a white spot in the presence of 100 and 500 µM of NaHS, respectively. No white spot was observed in oocytes treated either with 1 or 5 mM of NaHS. A close correlation between the white spot and the GVBD occurrence was confirmed by oocytes bisection after heat-fixation.

Seven hours after micro-injection, oocytes were individually analyzed by Western blotting. Two populations were discriminated: oocytes remaining in prophase I and oocytes in metaphase II. Microinjected oocytes exposed to low concentrations of NaHS and showing a white spot exhibited phosphorylated Erk2, p90^Rsk^ and dephosphorylated Cdk1 on tyrosine 15 (active MPF). Oocytes treated with higher concentrations of NaHS (1 and 5 mM), which did not exhibit a white spot, were observed to have a phosphorylation pattern identical to those of immature oocytes (Figure 5B). Thus, only the highest concentrations of NaHS were able to cause an inhibition of the meiosis resumption triggered by the micro-injection of mature oocytes cytoplasm.

### 3.6. NaHS Does Not Dissociate the Pre-MPF Complexes Nor Impact MPF Activity

**Figure 6:**
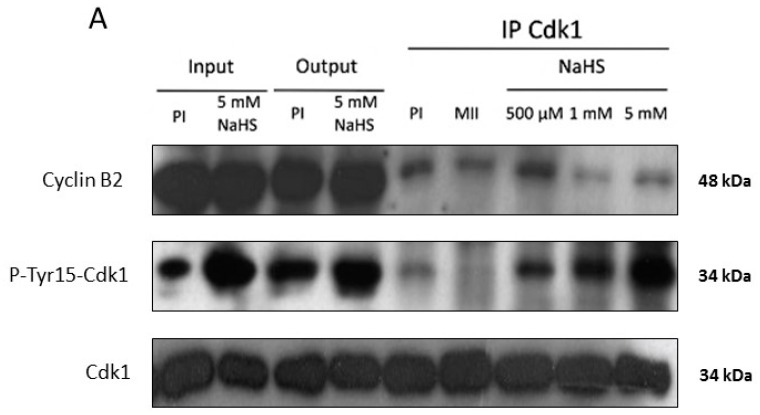

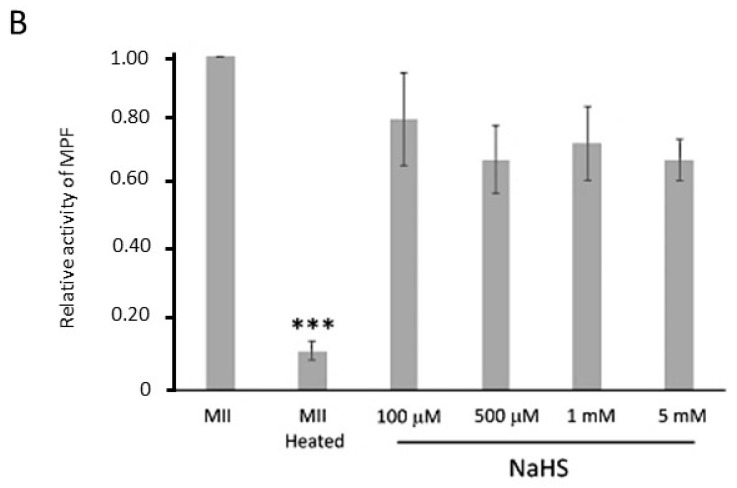
NaHS does not dissociate the pre-MPF complexes nor impact MPF activity. (**A**) Immunoblots of Cyclin B2, P-Tyr15-Cdk1, and total Cdk1 after Cdk1 immunoprecipitations. Protein extracts from immature oocytes (PI) were treated or not with NaHS at different concentrations (500 µM, 1 mM, and 5 mM) for 24 h at 4 °C. The protein extracts were incubated with Cdk1 antibody overnight before they were precipitated with Protein A-sepharose beads for 1 h at 4 °C. Input and output lines correspond to proteins extracts without the Cdk1 antibody but before and after protein A-sepharose bead incubation, respectively. MII corresponds to protein extracts from mature oocytes. (**B**) Relative MPF activity was examined by histone H1 kinase assay after exposure of protein extracts from mature oocytes (MII) to NaHS at different concentrations (100 µM, 500 µM, 1 mM, and 5 mM) or heated for 30 min at 100 °C. MPF activities correspond to the quantification values of *p*-histone H1 on H1 bands obtained by autoradiography on 3 independent experiments. Statistical significance was accepted for *** *p* < 0.001. N = 3; n = 15.

Since the MPF auto-amplification loop was altered, we further assessed the effects of NaHS on MPF. We first checked if NaHS could dissociate the pre-MPF complexes. Immunoprecipitation of Cdk1 and immunoblotting of cyclin B2 and P-Tyr15-Cdk1 were carried out on protein extracts from immature oocytes treated or not with NaHS at different concentrations. The signal corresponding to cyclin B2 revealed by immunoblotting indicates that MPF was not dissociated in presence of NaHS regardless of the used concentration (Figure 6A). The amount of tyrosine 15 phosphorylated Cdk1 is increased by NaHS in a dose-dependent manner suggesting that Cyclin B2 is associated with the inactive form of Cdk1. Since no pre-MPF dissociation occurred in presence of NaHS, the action of NaHS on meiosis resumption could be explained by direct inhibition of the MPF kinase activity. The MPF activity was further tested in a specific Cdk1 assay using histone H1 as a substrate. Protein extracts from mature oocytes (containing active MPF) were treated or not for 1 h with various concentrations of NaHS. A boiled control had a significantly reduced MPF activity (*p* < 0.001), whereas, regardless of the used concentration, the presence of NaHS had no significant effect on MPF activity (Figure 6B).

### 3.7. NaHS Impairs Human Cdc25C Activity In Vitro

**Figure 7:**
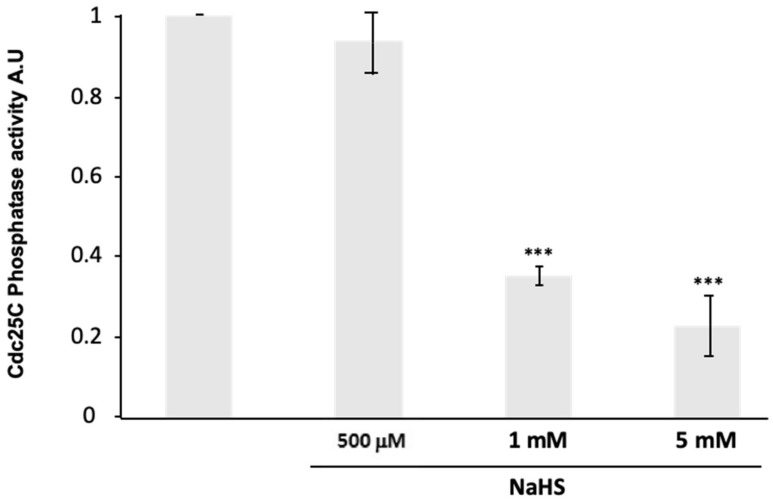
NaHS impairs human Cdc25C activity in vitro. The reactions were performed using 1 µg of human recombinant Cdc25C pre-incubated or not with NaHS (500 μM, 1 mM, or 5 mM). Results are shown as relative ratios to control without NaHS. Statistical significance was accepted for *** *p* < 0.001. Three independent experiments were performed.

Since NaHS had no direct effect on MPF, we further assessed the effect on one of its upstream regulators, the phosphatase Cdc25C. The activity of the human Cdc25C, treated or not for 15 min with NaHS at various concentrations, was tested in vitro using 3-O-methylfluoresceinphosphate as a substrate. Cdc25C activity showed a significant decrease (*p* < 0.001) by treatments with 1 and 5 mM of NaHS (Figure 7). The concentration of 500 µM of NaHS did not affect Cdc25C phosphatase activity compared to the control value.

### 3.8. NaHS Effects Are Partially ROS-Dependent

**Figure 8:**
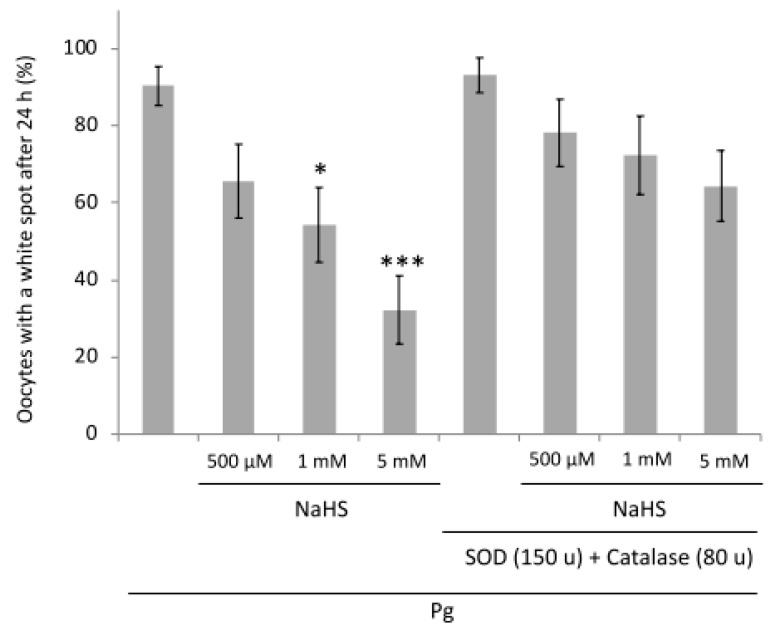
NaHS effects on meiosis resumption are reversed by ROS scavengers. Percentage of oocytes with a white spot 24 h after addition of progesterone (4 μg/mL). Oocytes were pre-incubated (or not) in the presence of NaHS at different concentrations and rescued with supplementation by SOD (150 units) and catalase (80 units). Statistical significance was accepted for * *p* < 0.05 and *** *p* < 0.001. N = 3; n = 15.

To determine whether the effects of NaHS on *Xenopus* meiosis resumption could be related to ROS (reactive oxygen species), as previously reported [39,40], the ROS dependent effects were counteracted by the use of antioxidant enzymes, superoxide dismutase (SOD), and catalase [38], respectively, used at 150 and 80 units. SOD and catalase used alone had no effect on meiosis resumption, as previously reported [41]. There was no difference between the progesterone stimulated oocytes treated or not with the antioxidant enzymes. After 24 h, control oocytes exhibited 90% of white spots against 93% in the presence of SOD and catalase (Figure 8). NaHS treatments resulted in a significant decrease of the oocyte percentages entering the M-phase (1 mM: *p* < 0.05; 5 mM: *p* < 0.001). The percentages of oocytes exhibiting a maturation spot in the conditions containing NaHS at concentrations of 500 µM, 1 mM, and 5 mM were 66%, 54%, and 32%, respectively. The decreased number of oocytes entering the M-phase after a treatment with NaHS was counteracted by SOD and catalase. No significant decrease of the white spot occurrence percentage was observed in presence of SOD and catalase when NaHS was applied: 78%, 72%, and 64% of the oocytes showed a maturation spot in the conditions containing 500 µM, 1 mM, and 5 mM of NaHS, respectively. The lack of significant statistical difference between SOD, catalase, and NaHS compared to control progesterone treated oocytes suggested that the effects of NaHS in *Xenopus* oocytes meiosis resumption were related to ROS effects.

### 3.9. NaHS Does Not Promote Nor Protect from Apoptosis

**Figure 9:**
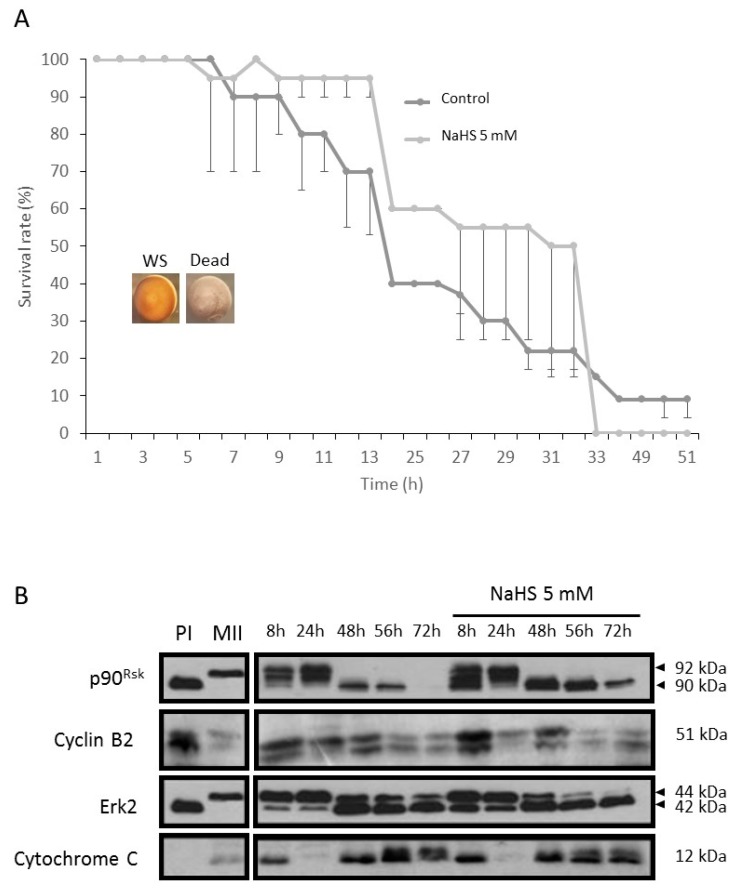
NaHS has no effect on apoptosis in *Xenopus* oocytes. (**A**) Survival rates of mature (WS) oocytes in presence or absence (control) of NaHS at the concentration of 5 mM. The number of dead oocytes was determined every hour. Microphotographs of healthy (WS) or dead (Dead) oocytes are shown. (**B**) Western blot of p90^Rsk^, Erk2, cyclin B2, and cytochrome C. Mature oocytes were incubated (or not) in NaHS at 5 mM for 8, 24, 48, 56, and 72 h. Some oocytes were maintained in culture medium without treatment (PI) or in progesterone (4 μg/mL) alone (MII).

Considering that the rise in the ROS level is correlated with an increase in apoptosis of *Xenopus laevis* oocytes blocked in metaphase II [42,43], NaHS could affect MII oocyte survival. After maturation completion, oocytes were submitted to a 5-mM NaHS treatment and the morphological apoptosis characteristics were observed. After 48 h, all the oocytes exhibited no characteristic signs of apoptosis (numerous white dots and holes at the animal hemisphere, fading of both vegetal and animal hemispheres and oocyte collapse [35,44]) (Figure 9A). To confirm these phenotypical observations, proteins involved in apoptosis were studied by immunoblotting. In both treated and untreated conditions, a loss of phosphorylation of the protein p90^Rsk^ was observed after 48 h. Only the control condition showed a total loss of p90^Rsk^ protein. Cyclin B2 was degraded at the same rate and the amount of cytochrome C generated by mitochondria during apoptosis increased similarly over time (Figure 9B). These experiments demonstrated that the increase of H_2_S, using NaHS does not potentiate nor delay death by apoptosis in MII oocytes.

## 4. Discussion

Few studies have attempted to untangle the role of H_2_S in reproductive tissues by targeting gametes. Among the three enzymes (CBS, CSE, and MPST) involved in the H_2_S metabolism, CBS and CSE have been detected in the female reproductive system as well as in placental and fetal tissues [8,9]. The effects of H_2_S in these tissues were thought to be mainly related to vasodilatation [45] or hypoxia (in the case of preeclampsia in the placenta [8,46]). In the present study, we took advantage of the amenability of *Xenopus laevis* oocytes to assess the role of hydrogen sulfide (H_2_S) as a gaseous signaling molecule in female gametes meiosis.

Our results highlighted an effect of H_2_S in the mechanisms regulating meiosis resumption in *Xenopus* oocytes stimulated by progesterone. The measurement of H_2_S concentrations in living cells is difficult to perform based on the volatile feature and the rapid half-life of the compound. Though H_2_S intracellular concentrations were not measured, endogenous H_2_S production could be detected in prophase I oocytes. Meiosis progression of oocytes into metaphase II proceed through a significant decrease in H_2_S production 3 h after progesterone addition while MPF is not activated (no phosphorylation of target histone H3: Supplementary Figure S3A). Impairing the metabolism of H_2_S and increasing H_2_S levels suggested that H_2_S could play a role at the physiological level in the modulation of oocyte meiosis. CBS, CSE, and MPST were detected in oocytes arrested in prophase I or in metaphase II. A joint inhibition of CBS, CSE, and MPST drove a significant drop by 10-fold of the H_2_S production and a clear acceleration of meiosis. An H_2_S donor prevented the G2/M transition in *Xenopus* oocytes in a dose-dependent manner when induced by progesterone. In oocytes exposed to a medium containing NaHS, the most commonly used H_2_S donor, a significant increase in H_2_S production was detected with an increase by 3.8-, 6.5-, and 10-fold for 500 μM, 1 mM, and 5 mM, respectively. NaHS is a fast H_2_S releasing donor in the first hour, which was used as a loading period before oocytes meiosis progression was stimulated by progesterone. The significant decrease in GVBD rate correlated with the highest concentrations strengthen the idea that H_2_S at high doses acts as an inhibitor of meiosis.

To circumscribe the mechanisms sensitive to H_2_S in *Xenopus* oocytes, several hypotheses were tested based on the premise that protein translation and MPF activation were necessary for proper meiosis mechanisms. Notably, we took advantage of the amenability of the *Xenopus* oocytes as a system for heterologous expression; we micro-injected tagged-Myc mRNAs and tested their translation in the presence of an H_2_S donor. In NaHS conditions, little or no accumulation of tagged-Myc proteins in prophase I oocytes were detected. In addition, under progesterone and NaHS treatment (NaHS 500 μM, 1 mM, and 5 mM), endogenous Mos protein was not translated, showing H_2_S acted at the translational machinery level, which has not previously been reported.

In order to determine whether NaHS may impair meiosis through an action on the MPF auto-amplification loop, we used mature metaphase II-arrested (MII) oocyte cytoplasm since the latter drives a meiotic resumption independent of protein translation [47,48,49]. Micro-injection of mature oocyte cytoplasm activates a self-amplification loop that embeds MPF and Cdc25c together [50,51,52]. When oocytes were micro-injected with cytoplasm from MII oocytes, only the highest concentrations of NaHS impaired GVBD. The H_2_S effect did not involve a separation of the MPF partners, cyclin B2 and Cdk1 [53,54], when exposed to NaHS. Moreover, H_2_S did not affect the MPF enzymatic activity itself, based on the histone H1 kinase assay suggesting H_2_S is likely to act upstream of MPF regulators. We similarly tested the effects of NaHS on MAPK activity but did not observe any effect (Supplementary Figure S1). Thus, H_2_S is likely to act on upstream MPF on its regulators, rather than on MPF itself. Despite the fact that NaHS was previously reported to increase cAMP levels in *Xenopus* oocytes [55], the absence of effects of the PKA inhibitor H-89 discarded this hypothesis as an upstream target (Supplementary Figure S2).

The meiotic maturation induced by a cytoplasmic transfer of MPF activated by the auto-amplification loop was more efficiently abolished by NaHS treatments (1 and 5 mM) compared to progesterone induction. We can suppose the signaling cascades upstream of MPF in prophase 1 receiving oocytes that were not treated by progesterone, did not decrease their endogenous H_2_S production, and probably did not bypass an endogenous necessary releasing effect. These results are in favor of a potential role for the natural H_2_S decrease occurring in prophase I oocytes under progesterone in meiotic resumption. The inhibitory effects of NaHS on meiosis resumption, whether it is induced by progesterone or by a cytoplasm transfer, could be explained by a default in the inhibition of the kinase Myt1 and/or the activation of the phosphatase Cdc25, two upstream regulators of the Cdk1–cyclin B stock. Cdc25C activity, measured using an in vitro phosphatase assay, significantly decreases under 1 and 5 mM of NaHS exposure and could explain the inhibitory effect of H_2_S on the MPF activity.

H_2_S-derived post-translational modification points to another possible action of H_2_S. H_2_S engages in covalent reactions with proteins and modulates their structural properties and functions. S-sulfhydration-H_2_S addition to cys—thiol or persulfide formation (-SSH)—affects a large variety of proteins [56,57]. Among them, phosphatases PTP1B in blood cells 93T [58] and Cdc25C in breast cancer cells [59] have been reported to be subjected to S-sulfhydration. S-sulfhydration of Cdc25C could be responsible for the inactivation of this phosphatase and the subsequent impairment of the MPF auto-amplification loop. This hypothesis is corroborated by an increased phosphorylation of Cdk1 tyrosine 15 residue in a dose-dependent fashion by NaHS treatment that could reflect the inactivation of Cdc25, which could favor the inhibitory kinase of MPF, Myt1. Two more piece of evidence strengthen this hypothesis: (i) while it is accepted that S-nitrosylation and S-sulfhydration occur on the same proteins, S-nitrosylation of Cdc25C has been identified [60,61,62], (ii) a Cdc25 crucial cysteine residue is inactivated by ROS [63].

The cellular redox status is determined by the balance between reactive oxygen species (ROS) production through the electron transport chain and the NADPH oxidase system, and sequestration, which is achieved by glutathione and thioredoxin. ROS affects many proteins, including those involved in the cell cycle, through the genomic pathway [64], which is not active in *Xenopus* oocytes. However, this pathway is not likely to be involved in the effects of NaHS in *Xenopus* oocytes, which enter the M-phase through non-genomic mechanisms because the DNA damage response (DDR) is not effective prior to the mid-blastula transition in embryos [65]. Though the increase in ROS has been associated with apoptosis in *Xenopus* oocytes [42,43], we did not observe such a correlation. SOD and catalase, which are known to counteract the ROS-dependent effects [38,66], significantly reduced the effects of endogenous H_2_S to the level of the controls treated by progesterone. This observation would suggest that the effect of H_2_S signaling involves ROS action. However, we previously stated that H_2_0_2_ (a ROS donor) does not prevent GVBD [40]. In addition to ROS, an action of the reactive sulfur species (RSS) generated by H_2_S could be considered in NaHS-treated oocytes [67]. Catalase microinjections in *Xenopus* oocytes seem to be leading to RSS elimination before ROS creation [68].

H_2_S has been mostly involved in embryo implantation and oocyte aging. Regarding meiosis resumption, the depletion of CBS by siRNA in mice oocytes was shown to prevent this process, but the siRNA was done in granulosa cells and not directly in oocytes. In porcine oocytes, while the main characteristics of meiosis appeared to be similar to *Xenopus* oocytes (dependence upon protein synthesis, MPF and MAPK pathways activation, meiotic resumption independent of MAPK activation [69]), responses towards perturbations driven by H_2_S differ. CBS, CSE, and MPST were detected in immature and mature *Xenopus* oocytes, whereas these enzymes exhibit a different profile of expression in porcine oocytes regarding their maturation state [21]. The differences in the expression profiles between the two species offer a first insight into the difference of oocyte behaviors toward H_2_S modulation in vertebrates. Another difference lies in the fact that while the H_2_S donor offered protective effects towards aging in porcine oocytes [20], NaHS did not improve the survival rate of metaphase II-blocked *Xenopus* oocytes. However, such differences may also arise from the different nature of the H_2_S donor used [22].

In *Xenopus* oocytes, NaHS could block meiosis resumption in response to progesterone by negatively regulating protein synthesis and targeting the upstream regulators of MPF auto-amplification presumably by inhibition of Cdc25C. Further work will be necessary to characterize the proteins targeted by S-sulfhydration *in ovo* to untangle the nature of oocyte sensitivity towards the H_2_S gasotransmitter.

## Supplementary Materials

The following are available online at https://www.mdpi.com/2073-4409/9/1/237/s1, Figure S1: NaHS does not modify MAPK activity. Figure S2: NaHS effects on meiosis resumption induced by PKA inhibition. Figure S3: Immunoblots of total histone H3 (H3) and phosphorylated histone H3 (P-ser10-H3). Figure S4: Immunoblots of P-Tyr15-Cdk1 and total Cdk1 after Cdk1 immunoprecipitations.
