# Supplementary material for: Hydrogen Sulfide Impairs Meiosis Resumption in Xenopus laevis Oocytes
Source: Cells. 2020 Jan 17;9(1):237. doi: 10.3390/cells9010237 (PMC7017156; doi:10.3390/cells9010237)
Supplement: Supplementary file 1 [file cells-09-00237-s001.pdf]

Supplementary Materials

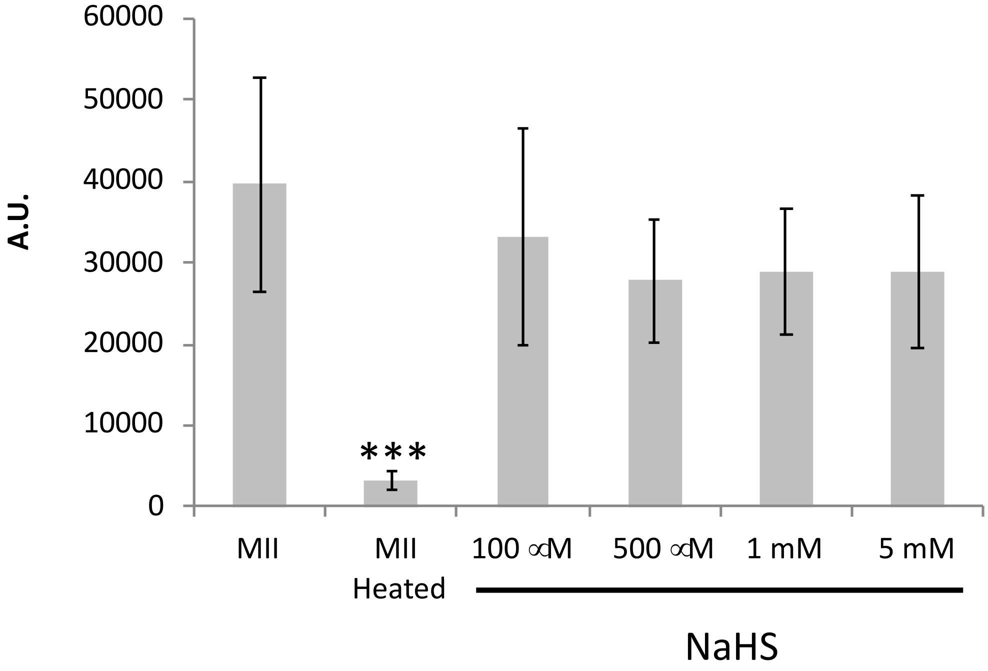

Figure S1. NaHS does not modify MAPK activity. MAPK activity was examined by a myelin basic protein (MBP) kinase assay on oocytes exposed to NaHS at 100 μM, 500 μM, 1 mM and 5 mM. Supernatants from mature oocytes (MII) were heated or not for 30 min at 100 °C before they were incubated with p9CKShs1–Sepharose beads (MPF–free) for 1h. Supernatants (5 μl) were incubated for 30 min at 30 °C with 1 volume of reaction buffer composed by 100 mM 3-[n-morpholino] propanesulfonic acid pH 7.2, 20 mM para-nitrophenyl phosphate, 40 mM β-glycerolphosphate, 20 mM $MgCl_2$, 10 mM EGTA, 0.2 mM EDTA, 5 μM cAMP-dependent protein kinase inhibitor, 2 mM benzamidine, 40 μg/mL leupeptin, 40 μg/mL aprotinin, 600 μM ATP, added with 3 mg MBP/mL) and 500 μCi/mL [γ-^{32}P]ATP. MAPK activities correspond to the quantification of P-MBP bands on the autoradiography. Statistical significance was accepted for *** $p < 0.001$. N = 3; n = 15.

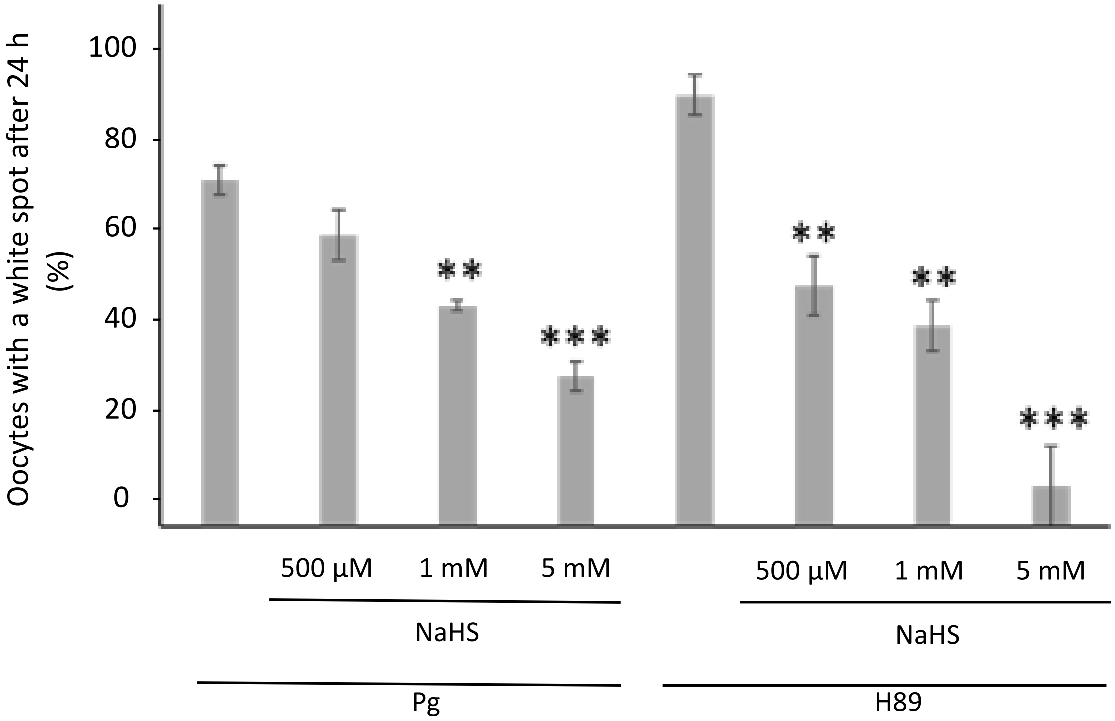

Figure S2. NaHS effects on meiosis resumption induced by PKA inhibition. Percentage of oocytes pre-incubated (or not) with NaHS at different concentrations and displaying a white spot 24h after addition of progesterone (4 µg/mL) or H-89 (100 µM). Statistical significance was accepted for **p<0.01 and *** $p < 0.001$. N = 3; n = 15.

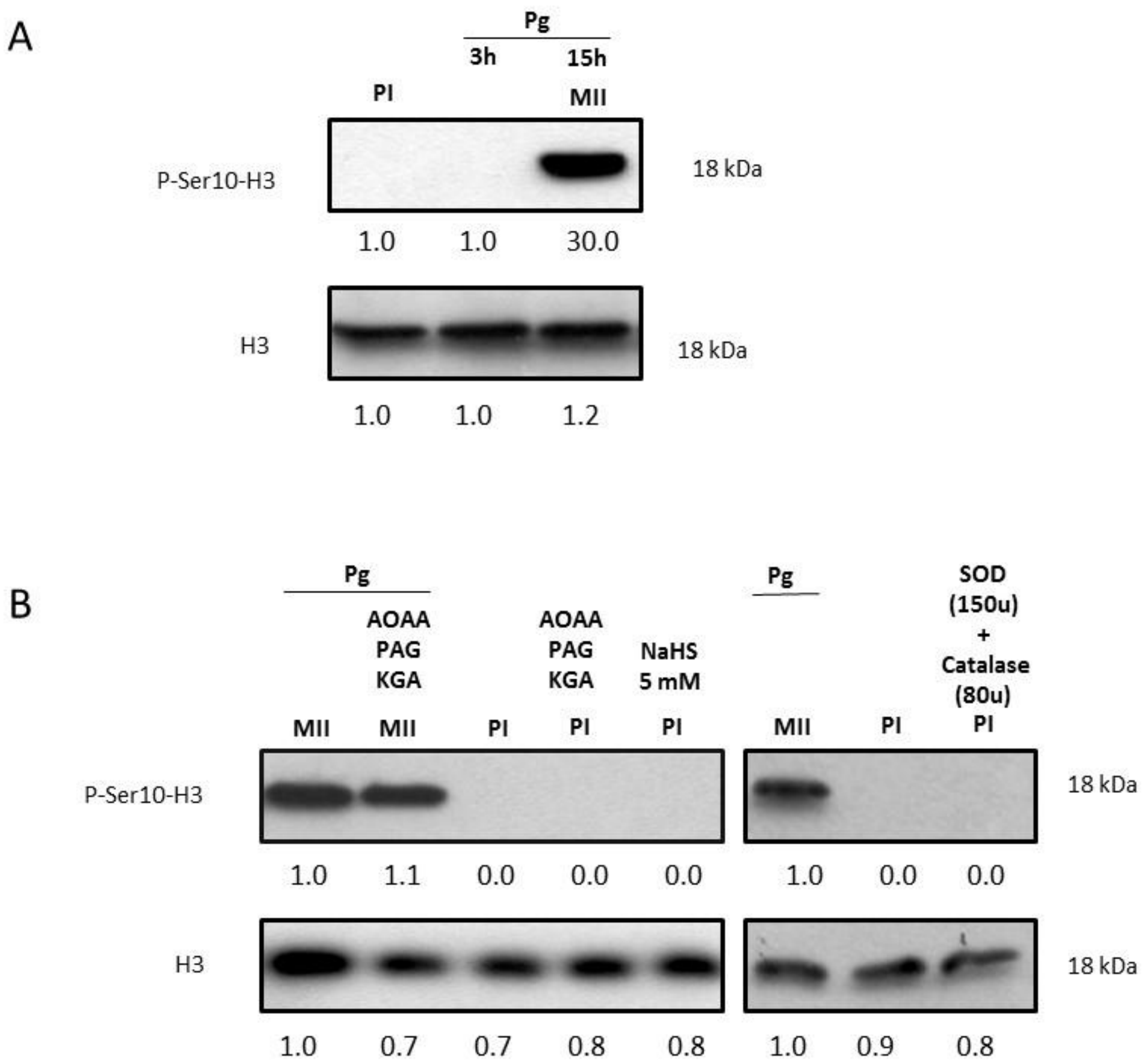

Figure S3. Immunoblots of total histone H3 (H3) and phosphorylated histone H3 (P-ser10-H3), A/ in prophase I (PI) oocytes, and in 3 and 15 h progesterone (4 µg/mL) treated oocytes (Pg 3 h and Pg 15 h). A pool of ten oocytes was lysed and proteins extracted and submitted to Western blot analysis as described in the materials and methods section. Relative intensity of P-ser10-H3 bands were normalized with the intensity of the loading control total H3 bands and related to the intensity of the control PI oocytes. P-ser10-H3 and H3 bands intensity were determined using Image J software. B/ Immunoblots of total histone H3 (H3) and phosphorylated histone H3 (P-ser10-H3) in metaphase II (MII) and prophase I (PI) oocytes. Oocytes were pre-incubated or not with H_2S-releasing enzyme inhibitors (AOAA (aminooxyacetic acid-10 mM), PAG (DL-propargylglycine-10 µM), and KGA (ketoglutaric acid-10 µM)) or with 5 mM of NaHS or with ROS scavengers SOD (150 units) and catalase (80 units) before stimulation or not by progesterone (Pg 4 µg/mL). Some oocytes were maintained in the culture medium without or with progesterone (Pg 4 µg/mL) treatment alone. After 15h, oocytes were lysed and proteins extracted and submitted to Western blot analysis as described in the materials and methods section. Relative intensity of P-ser10-H3 bands were normalized with the intensity of the loading control total H3 bands and related to the intensity of the control MII treated with progesterone. P-ser10-H3 and H3 bands intensity were determined using Image J software.

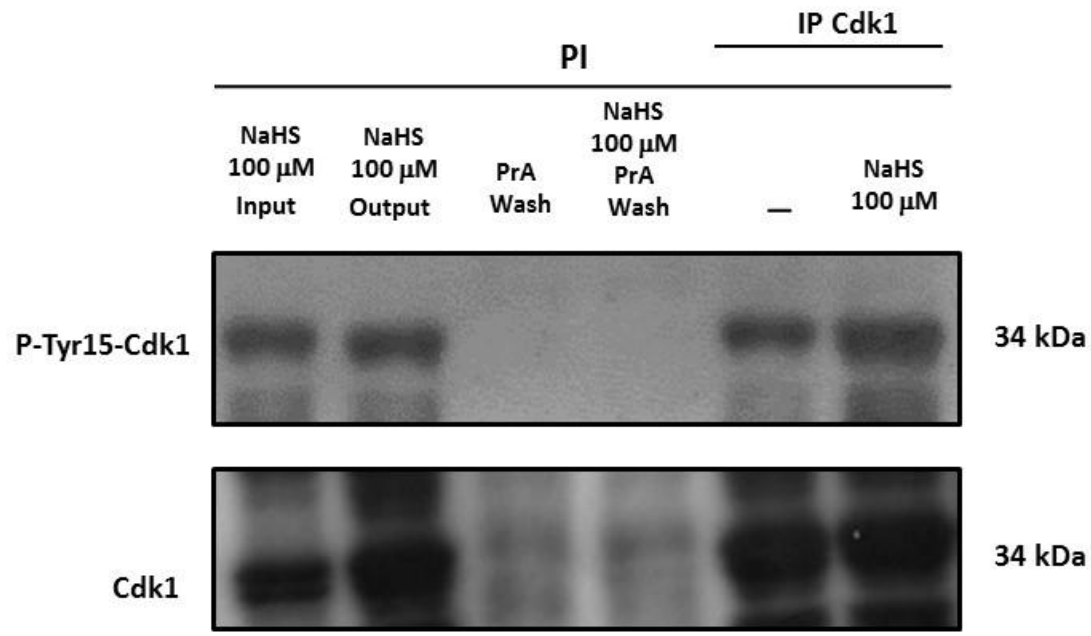

Figure S4. Immunoblots of P-Tyr15-Cdk1 and total Cdk1 after Cdk1 immunoprecipitations. Protein extracts from immature oocytes (PI) were treated or not with NaHS at 100 µM for 2 h at 4 °C and washed with Protein A-Sepharose beads for 1 h at 4 °C (PrA wash). The protein extracts were incubated with Cdk1 antibody overnight before they were precipitated with Protein A-Sepharose beads for 1 h at 4 °C. Input and output lines correspond to proteins extracts without Cdk1 antibody but respectively before and after Protein A-Sepharose beads incubation. PrA wash lines correspond to proteins fixed on Protein A-Sepharose beads after the first wash of extracts.
